# ZNF143 in Chromatin Looping and Gene Regulation

**DOI:** 10.3389/fgene.2020.00338

**Published:** 2020-04-07

**Authors:** Bingyu Ye, Ganggang Yang, Yuanmeng Li, Chunyan Zhang, Qiwen Wang, Guoying Yu

**Affiliations:** ^1^State Key Laboratory of Cell Differentiation and Regulation, Henan Normal University, Xinxiang, China; ^2^Henan International Joint Laboratory of Pulmonary Fibrosis, Henan Normal University, Xinxiang, China; ^3^Henan Center for Outstanding Overseas Scientists of Pulmonary Fibrosis, Henan Normal University, Xinxiang, China; ^4^College of Life Sciences, Henan Normal University, Xinxiang, China; ^5^Institute of Biomedical Science, Henan Normal University, Xinxiang, China; ^6^Overseas Expertise Introduction Center for Discipline Innovation of Pulmonary Fibrosis (111 Project), Henan Normal University, Xinxiang, China

**Keywords:** transcription factor, ZNF143, biomarker, chromatin organization, loop

## Abstract

ZNF143, a human homolog of the transcriptional activator Staf, is a C2H2-type protein consisting of seven zinc finger domains. As a transcription factor (TF), ZNF143 is sequence specifically binding to chromatin and activates the expression of protein-coding and non-coding genes on a genome scale. Although it is ubiquitous expressed, its expression in cancer cells and tissues is usually higher than that in normal cells and tissues. Therefore, abnormal expression of ZNF143 is related to cancer cell survival, proliferation, differentiation, migration, and invasion, suggesting that new small molecules can be designed by targeting ZNF143 as it may be a good potential biomarker and therapeutic target for related cancers. However, the mechanism on how ZNF143 regulates its targeting gene remains unclear. Recently, with the development of chromatin conformation capture (3C) and its derivatives, and high*-*throughput sequencing technology, new findings have been obtained in the study of ZNF143. Pioneering studies have showed that ZNF143 binds directly to promoters and contributes to chromatin interactions connecting promoters to distal regulatory elements, such as enhancers. Further, it has proved that ZNF143 is involved in CCCTC-binding factor (CTCF) in establishing the conserved chromatin loops by cooperating with cohesin and other partners. These results indicate that ZNF143 is a key loop formation factor. In addition, we report ZNF143 is dynamically bound to chromatin during the cell cycle demonstrated that it is a potential mitotic bookmarking factor. It may be associated with CTCF for mitosis-to-G1 phase transition and chromatin loop re-establishment in early G1 phase. In the future, researchers could further clarify the fine mechanism of ZNF143 in mediating chromatin loops with the help of CUT&RUN (CUT&Tag) and Cut-C technology. Thus, in this review, we summarize the research progress of TF ZNF143 in detail and also predict the potential functions of ZNF143 in cell fate and identity based on our recent discoveries.

## Introduction

Schuster et al. (1995) found a transcription factor (TF), which can be bound specifically to the promoter of selenocysteine tRNA in *Xenopus* oocytes and named it Staf (selenocysteine tRNA gene transcription activating factor). In the same year, Tommerup and Vissing (1995) reported zinc finger protein 143 (ZNF143), a human homolog of the transcriptional activator Staf, was located on the human 11th chromosome, 11p15.3–15.4. Subsequently, Adachi et al. (1998) isolated and characterized m*-*Staf from mouse mammary gland, which is consistent with human ZNF143. ZNF143 is a member of the Kruppel family and is a widely expressed transcriptional activation factor that regulates gene expression associated with cell cycle and DNA replication (Izumi et al., 2010). Therefore, it is widely involved in a variety of cellular and pathogenic processes, such as cell survival, growth, proliferation, etc. (Table 1). However, the molecular mechanism of ZNF143 in regulating gene expression remains elusive.

**TABLE 1:** The role of ZNF143 in cancer progression.

ZNF143 status	Cancer type	Association	References
Knockdown	Human prostate cancer PC3	Induce cell apoptosis	Izumi et al., 2010
	Breast carcinoma	Increase cellular motility	Paek et al., 2017
	Colon cancer (HCT116)	Increase cell migration and invasion	Paek et al., 2014
	HeLa-S3	Reduce cell proliferation	Ngondo-Mbongo et al., 2013
	Breast cancer	Better cell survival	Paek et al., 2019
	HeLa	Reduce cell proliferation, cell-cycle progression, and cell viability	Parker et al., 2014
	Colon cancer	Increase cell plasticity	Verma et al., 2019
Overexpression	PC3 prostate cancer cell lines	Increase cell division	Izumi et al., 2011
	Gastric cancer(GC)	Enhance GC migration	Wei et al., 2016
	HepG2 and HeLa	Increase cell survival and differentiation	Grossman et al., 2014
Positively expression	Lung cancer	Increase cell growth	Kawatsu et al., 2014
	Lung adenocarcinoma	With highly invasive and proliferation	Kawatsu et al., 2014
	Ovarian tumors and Low-grade ovarian cancers	Relate to cancer invasion, metastasis formation	Sadlecki et al., 2019

In recent years, studies have revealed that ZNF143 not only exists in most cancer cells but is also necessary for the normal development of tissues (Izumi et al., 2011; Halbig et al., 2012; Kawatsu et al., 2014; Paek et al., 2014; Wei et al., 2016; Paek et al., 2017). Genome-wide analyses have shown that TF ZNF143 with sequence binding specificity is usually bound to the promoter of its regulatory gene and promotes the formation of chromatin loop by interacting with other chromatin structure and organization factors, such as CCCTC-binding factor (CTCF) and cohesin (Heidari et al., 2014; Bailey et al., 2015; Ye et al., 2016; Yang et al., 2017; Mourad and Cuvier, 2018; Wen et al., 2018). In summary, as a key TF, ZNF143 plays a critical role in chromatin loop formation and gene regulation (Table 2), illustrating great importance in the study of its regulatory mechanism.

**TABLE 2:** ZNF143 plays a critical role in chromatin interaction.

Cell type	Detection method	Interaction factor	References
GM12878, K562, HelaS3	Carbon-copy chromatin conformation capture (5C), 3C, ChIP-seq	Cohesin (SMC3), CTCF	Bailey et al., 2015
GM12878, K562	ChIA-PET, ChIP-seq, RNA-seq	Cohesin (RAD21), CTCF	Heidari et al., 2014
GM12878, K562	ChIA-PET, ChIP-seq	Cohesin (RAD21 and SMC3), CTCF	Ye et al., 2016
Kc167, GM12878	Hi-C, ChIP-seq	Cohesin (RAD21), CTCF	Mourad and Cuvier, 2018
HEK293T	Hi-C	Cohesin (RAD21), CTCF	Wen et al., 2018
HeLa-S3, HEK293, K562, HPB-ALL, NIH3T3, mESC, MEF	ChIP-Seq, RNA-seq	Notch1, THAP11	Ngondo-Mbongo et al., 2013
293T/17, HeLa, SW620, T98G	ChIP-Seq	THAP11, HCF-1	Parker et al., 2014; Vinckevicius et al., 2015
Mouse ES	ChIP	Oct4	Chen et al., 2008
Human TLL	RNA-microarray, ChIP-Seq	Notch1, RBPJ	Wang et al., 2011
HeLa	RNA-microarray, ChIP-Seq	HCF-1, THAP11, YY1, GABP	Michaud et al., 2013

## The Structural Features of ZNF143

**FIGURE 1:**
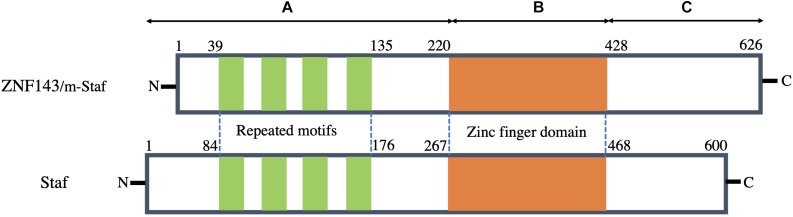
Schematic representation of structural features of human ZNF143 in comparison with m-Staf and Staf. Three regions of these three proteins can be distinguished: A contains mRNA and snRNA activation domains with the presence of the four repeated motifs. B is the central seven zinc finger domain and is therefore a DNA binding domain. C is a region with unknown function.

The amino acid sequence of human ZNF143 is highly homologous to both m*-*Staf and Staf. Among its sequence, 97.1 and 84% residues are identical to those of m-Staf and Staf, respectively (Schuster et al., 1995; Adachi et al., 1998; Myslinski et al., 1998). Structurally, these proteins consist of three regions (A, B, and C) (Figure 1). Analysis of the three regions indicates that the central region B (residues 220–428 in ZNF143 and m*-*Staf, residues 267–468 in Staf) encompasses seven tandemly repeated zinc fingers of the C2H2 type, is highly basic, while the regions A (residues 1–219 in ZNF143 and m*-*Staf, residues 1–266 in Staf) encodes four repeated motifs and C (residues 429–626 in ZNF143 and m*-*Staf, residues 469–600 in Staf) are acidic (Figure 1). The central region of seven zinc fingers domain is the DNA binding domain. Outside of the central domain, N-domain (region A) is the activation domain both for mRNA and snRNA, and the characteristic features of this domain of these three proteins are very simlar. The function of C-domain (region C) is unclear (Myslinski et al., 1998). Strikingly, the four repeated motifs can be observed between residues 39 and 135 in region A of ZNF143/m-Staf (residues 84 and 176 in region A of Staf) (Figure 1). Each repeat motif contains 15 amino acids and the distance between them contains 10–12 amino acids (Schuster et al., 1995).

As a TF, it is noted that the tandemly repeated zinc finger domain (DNA binding domain) and the element of repeated motifs (activation domain) are especially well conserved among these three proteins (Myslinski et al., 1998). It is reported that this TF possesses the capacity to bind over 2000 promoter regions of both mRNA and small nuclear RNA (snRNA) genes (Myslinski et al., 1998, 2006). Recently, Ngondo-Mbongo et al. (2013) have found that ZNF143 has two main DNA binding motifs of high affinity, namely, SBS1(GTTATGGAATTCCCATTATGCACCGCG) and SBS2 (AAACTACAATTCCCATTATGCACCGCG). Both of them are closely related to its specific binding on the chromatin, and thus initiate gene expression and regulation.

## The Function of ZNF143

### Regulating Cell-Cycle Progression

TF ZNF143 regulates gene expression associated with cell cycle. Many studies utilize knockdown or overexpression methods to evaluate the effect of ZNF143 on cancer cell progression. For example, Izumi et al. (2010) have reported that ZNF143 is associated with cell cycle and cell proliferation, whereas ZNF143 knockdown causes human prostate cancer PC3 cells to stagnate during G2/M and is accompanied with apoptosis. By establishing two forced expression of ZNF143 PC3 cancer cell lines, they found that overexpress genes strongly associated with cell cycle and cell division (Izumi et al., 2011). ZNF143 knockdown induces increased breast cancer motility, which indicates that ZNF143 expression contributes to breast cancer progression (Paek et al., 2017). In addition, low ZNF143 expression exhibits better cell survival through an autophagic process by regulating the p53–Beclin1 axis in breast cancer cells (Paek et al., 2019). ZNF143 is essential and sufficient for Skp2 promoter activity and ZNF143 silencing inhibits cell proliferation; however, ectopic ZNF143 can rescue Skp2 expression (Hernandez-Negrete et al., 2011). Overexpression of ZNF143 enhances transaldolase promoter activity in HepG2 and HeLa cells and ZNF143 plays a key role in controlling cell survival and differentiation (Grossman et al., 2014). Simultaneously, other researchers have reported that THAP11/ZNF143/HCF-1 complex is an indispensable component of the transcriptional regulatory network and disruption of this complex leads to reduced cell proliferation, cell-cycle progression, and cell viability (Parker et al., 2014). Ngondo-Mbongo et al. (2013) have also showed that ZNF143, ICN1, and THAP11 play a pivotal role in modulating cell proliferation of rapidly dividing cells. Myslinski et al. (2007) have found that human BUB1B gene mediates the activity of spindle checkpoints to ensure chromosomal stability and euploidy, requires ZNF143 binding.

### Regulating Embryonic Development and Maintaining Stem Cell Identity

As a key TF, ZNF143 has a critical function in regulating embryonic development. Halbig et al. (2012) have found that ZNF143 significantly changes zebrafish embryonic phenotypes. Therefore, ZNF143 is necessary for the normal development of zebrafish embryos. The identification and characterization of paralogous genes is also critical for understanding gene function. In the functional study of ZNF143, Huning and Kunkel (2020) have found that *znf143a*, a novel paralog of *znf143*, encodes a strong transcriptional activator protein and performs a similar role in the normal development of zebrafish embryos but expressed at a different level during early development. In mouse embryonic stem (ES) cells, ZNF143 regulates Nanog by regulating the binding of Oct4, and ZNF143 is also critical for maintaining human ES cell identity (Chen et al., 2008).

### Potential Drug Design Target

TF ZNF143 is a potential drug design target to treat solid cancers. After cisplatin treatment, the binding activity of ZNF143 and MRP S11 significantly increases. This indicates that ZNF143 is involved in response to DNA damage (Ishiguchi et al., 2004; Torigoe et al., 2005; Wakasugi et al., 2007). P73 promotes ZNF143 binding with cisplatin-modified DNA, indicating that ZNF143 can regulate the transcription of DNA repair genes (Wakasugi et al., 2007). ZNF143 can also mediate cell survival by upregulating glutathione peroxidase (GPX1) activity. Thus, ZNF143 interference can increase drug sensitivity to cisplatin treatment of mitochondrial dysfunction (Lu et al., 2012). GAIP-interacting protein, C-terminus (GIPC) induces ZNF143 expression by participating in IGF-1 signal transduction to regulate reactive oxygen products (Paek and You, 2011). ZNF143 is also involved in the migration and invasion of colon cancer cells through a ZEB1-E- cadherin-linked pathway (Paek et al., 2014). The expression levels of ZNF143 and IL-8 are inversely correlated with three-dimensionally grown spheroids and colon cancer tissues (Verma et al., 2019). ZNF143 is accompanied with an increase in MIB-1 index in patients with lung adenocarcinoma, leading to high cell proliferation activity and poor prognostic treatment (Kawatsu et al., 2014). Wei et al. (2016) have found that ZNF143 expression can enhance the metastasis of gastric cancer cells, indicating that ZNF143 can be a drug target for the treatment of gastric cancer. The reduction in ZNF143 expression eventually leads to the cobaltamine transport protein not effectively transporting cobalamin (Pupavac et al., 2016). The expression patterns of ZNF143 and ZNF281 in serous borderline ovarian tumors (SBOTs) and low-grade epithelial ovarian carcinomas (EOCs) play a key role in cancer invasion, metastasis formation, and chemotherapy resistance (Sadlecki et al., 2019). ZNF143 is an upstream regulator to increase the expression of the RNA binding protein TARBP2 in breast and lung cancers (Fish et al., 2019). Thus, how to effectively design small molecule drugs to target ZNF143 is imminent. Fortunately, Haibara et al. (2017) have found that new small molecules YPC-21661 and YPC-22026 can reduce the expression of their target genes RAD51, PLK1, and Survivin by inhibiting the binding of ZNF143 to their promoters. In the future, it is believed that more and more molecule drugs will be exploited by targeting ZNF143 to treat related cancers.

## ZNF143 Regulates Gene Expression and Its Mechanism

### ZNF143 Participates in the Regulation of Coding and Non-coding Genes

As an important TF, ZNF143 regulates the expression of various genes. During transcription activation, Schuster et al. (1998) have found that ZNF143 activation domains bound by mRNA and snRNA are different. Myslinski et al. (1998) first have found ZNF143 can activate the transcription from RNA polymerase II TATA box-containing mRNA promoters. For example, Kubota et al. (2000) have reported that ZNF143 is the key TF upregulating the molecular chaperone coding gene Cctα transcription through binding with the two activation elements (CAE1 and CAE2). Mach et al. (2002) have also showed that ZNF143 stimulates transcription of the human interferon regulatory factor-3 (IRF-3) gene by binding to *Sph*I postoctamer homology (SPH) elements *in vitro* and in transfected cells. ZNF143 plays an important role in the transcription of neuronal nitric-oxide synthase (nNOS) exon 1, the mutation of the binding site of ZNF143 leads to a significant reduction in the activity of this exon (Saur et al., 2002). Barski et al. (2004) use ChIP as well as deletion/mutation analysis reveal that the aldehyde reductase is significantly enhanced by transcription activation after binding to ZNF143. Di Leva et al. (2004) have found that ZNF143, together with CAAT factors, regulates human synaptobrevin-like 1 (SYP-like 1) through binding to the SYBL1 promoter in HeLa cells. Gerard et al. (2007) have reported that ZNF143 binds to the promoter of mitochondrial TF A (Tfam) to regulate transcription initiation and replication of mitochondrial DNA in consistent with Sp1, NRF-1, and NRF-2. ZNF143 binds with the −305/−107 of the BUB1B promoter to regulate BUB1B expression to maintain chromosomal stability and euploidy (Myslinski et al., 2007). Gonzalez et al. (2017) have reported that ZNF143, specifically binds to the 8-bp sequence (CCCAGCAG), ∼100 bases upstream of the C/EBPα transcription start site (TSS), plays an important role in the expression of C/EBPα in myeloid cells.

ZNF143 acts as a transcription-activated factor under the joint action of RNA polymerase III (Schaub et al., 1997). The snRNA and snRNA-type genes require the binding of ZNF143 during transcription, such as human U4C, U6, Y4, 7SK; mouse U6 RNAs and *Xenopus* U1b1, U2, U5, MRP. However, the binding of ZNF143 to snRNA occurs on a distal sequence element (DSE) (Schaub et al., 1997). By comparing ZNF143 recognition sequence of human U6 snRNA and selenocysteine tRNA, Schaub et al. have found that there are only 47% consistent in sequences. In the seven zinc fingers of ZNF143 recognition sequence, the first zinc finger is necessary for selenocysteine tRNA promoter identification, whereas U6 snRNA is not. The seventh zinc finger is essential for the binding activity of them. The flexibility binding results in differences in transcription activation mechanisms (Schaub et al., 1999a). U6 snRNA transcription activation requires ZNF143–DNA–Oct-1 complex, whereas selenocysteine tRNA requires ZNF143-DNA complex (Schaub et al., 1999b). Schaub et al. (2000) have found that zinc fingers 3–6 are the minimum zinc finger regions.

### Self-Regulation of ZNF143

To maintain stable ZNF143 expression at normal levels, the transcription feedback regulation mechanism is the simplest and most direct means. ZNF143 selectively adjusts reverse expression by using a low affinity binding site (TSS2) located downstream of the TSS. When ZNF143 expression is higher than normal, transcripts containing longer 5′-UTR (few translation products) are produced by TSS2 transcription. In addition, when ZNF143 levels are lower than normal, the canonical TSS1 binding site is used to express transcripts containing shorter 5′-UTR (many translation products). This transcriptional auto-regulatory mechanism regulates ZNF143 expression by the conversion of the TSS switch, which plays an important role in cell proliferation and growth (Ngondo and Carbon, 2004). Given that ZNF143 is closely related to many biological processes, its expression must be strictly regulated. Ngondo et al. have found that ZNF143 transcripts have three different lengths of 3′-UTR, with the longer 3′-UTR isoform containing variable polyadenylation sites, miRNA target sites, or AU-rich element (ARE). Thus, it tends to post-transcriptional regulation. The longest 3′-UTR isoform contains an unstabilizing ARE and is targeted by mir-590-3p. These results emphasize that ZNF143 post-transcriptional regulation depends on the long 3′-UTR isoform (Ngondo and Carbon, 2014).

### ZNF143 Is a Chromatin-Looping Factor

Myslinski et al. have predicted the whole genome binding sites of ZNF143 through computer simulation (*in silico*) and biochemical methods. They speculated that at least 2500 ZNF143-binding sites are distributed in 2000 promoter regions throughout the mammalian genome. Further research has found that the presence of ZNF143-binding site alone can initiate the expression of a luciferase reporter gene, suggesting that ZNF143 itself exhibits the ability to recruit the transcription machinery (Myslinski et al., 2006). Recently, Wang et al. have reported the co-localization of RBPJ/Notch1/ZNF143, in which ZNF143 can bind with 40% of the Notch1 sites, and RBPJ shows high promoter binding preference by embedding in the ZNF143 motifs. These results may indicate a dynamic exchange of RBPJ/Notch1 and ZNF143 complexes through competition in the binding sites (Wang et al., 2011). Ngondo-Mbongo et al. (2013) have revealed that ZNF143, THAP11, and Notch1 regulate the common target genes through the mutually exclusive occupation of overlapping binding sites. Michaud et al. (2013) have found that HCF-1 is bound with 5400 CpG island promoters. HCF-1, ZNF143, and THAP11 exhibit co-localization, with HCF-1 in collaboration with ZNF143 and THAP11 plays an important role in the transcriptional regulation of HeLa cells. Parker et al. have found that HCF-1, as a coregulator of the TF E2F proteins, is not directly collected in the promoter region but is mediated by ZNF143 and THAP11. HCF-1/ZNF143/THAP11 as a complex that occupies specific sites of chromatin co-regulates the expression of cell proliferation genes (Parker et al., 2014). However, how DNA sequences guide the THAP11/ZNF143/HCF-1 complex to chromatin remains in dispute. Vinckevicius et al. (2015) have explicitly proposed that ACTACA, as a joint submotif of ZNF143 and THAP11, guides THAP11 and HCF-1 to ZNF143-occupied loci and emphasized the importance of the position, spacing, and direction relative to the ZNF143 core motif.

TF ZNF143 can interact with other transcriptional regulators in mediating chromatin loop formation. Chromatin interactions between promoters and long-region regulatory elements can determine the expression level of a gene (Fraser, 2006; Fraser and Bickmore, 2007). In recent years, with the development of high-throughput sequencing and chromatin conformation capture technologies (3C, chromatin conformation capture; Hi-C, chromatin conformation capture using high throughput sequencing; ChIA-PET, chromatin interaction analysis by paired-end tag sequencing) (Dekker et al., 2002; Fullwood et al., 2009; Lieberman-Aiden et al., 2009), increasing evidence indicates that the interaction between genomic regulatory elements plays an important role in regulating gene expression. Heidari et al. (2014) have discovered that ZNF143 plays an important role in mediated distal chromatin interactions. Bailey et al. (2015) have found that ZNF143, as a novel and key chromatin-looping factor, with sequence specificity dependency at promoters and links the distal regulatory elements together, playing an important role in the establishment of the genomic organization. ZNF143 binds to the PMM2 promoter could establish a functional chromatin loop enabling interaction between the promoter and distal regulatory elements, which allows specific spatiotemporal regulation of PMM2 (Cabezas et al., 2017). ZNF143 knockdown mainly eliminates or destabilizes chromatin loops (Wen et al., 2018). We also found that ZNF143 was involved in the CTCF-mediated chromatin interactions by cooperating with cohesin (Ye et al., 2016). Other researchers have showed that ZNF143 interactes with other regulators are also important for chromatin domain formation. For example, Mourad and Cuvier (2006) have revealed that the formation of 3D chromatin domains is affected by positive driving factors CTCF, cohesin, ZNF143, polycomb proteins, and negative driving factors P300, RXRA, BCL11A, ELK1. CTCF binding sites are not only closely associated with topologically associating domain (TAD) boundaries, but also interact with ZNF143 and Yin Yang (YY)1 (Hong and Kim, 2017).

## Conclusion and Prospects

ZNF143 can bind with multi-species, multi-type coding and non-coding genes (Schuster et al., 1995; Schaub et al., 1997; Myslinski et al., 1998). However, ZNF143 binding and co-initiative transcription differs due to the diversity of promoter structures. Although the promoter structure of H1 RNA, the RNA component of the human nuclear RNase P, is similar to that of vertebrate snRNA, H1 RNA’s promoter is distributed within 100 bp of the 5′ flanking sequence and presents a highly compact structure to initiate transcription (Myslinski et al., 2001). ZNF143 binding with U6 found in zebrafish are located upstream of the TATA box and downstream of proximal sequence element (PSE), unlike the U6 of other species (Halbig et al., 2008). The promoter of SCARNA2 is contained within 161 bp upstream of TSS due to its special transcription (different from SCARNA), whereas ZNF143 is the basic regulator (Gerard et al., 2010).

As a general TF, ZNF143 participates in numerous cellular biological activities. Using comparative genomic analysis to identify the distribution of ZNF143 target genes, Myslinski et al. (2006) have found that DNA binding and TFs account for 23%, protein synthesis/degradation/modification account for 21%, and DNA replication/cell cycle/cell growth/differentiation/apoptosis account for 13%. Anno et al. have also found that ZNF143 *per se* exhibits an inherently bidirectional transcription activity. Thus, ZNF143 has the ability to control the expression of divergent protein–protein and protein–non-coding RNA gene pairs (Anno et al., 2011). ZNF143 is expressed differently in various tissues. It is highly expressed in the lung, ovary and thymus, but weakly expressed in the brain, liver, and kidney (Grossman et al., 2014). ZNF143 is highly expressed in many solid tumors, and it is involved in cisplatin resistance because cisplatin induced ZNF143 binds to cisplatin-modified DNA (Wakasugi et al., 2007; Paek and You, 2011; Lu et al., 2012). Thus, novel small molecules can be designed for ZNF143 to enhance the sensitivity of cisplatin chemotherapy (Haibara et al., 2017). ZNF143 is not only indispensable for the embryonic development of zebrafish but also necessary for ES cell identity and self-renewal capability of ES cell (Chen et al., 2008; Halbig et al., 2012). What is more, histone methylation in the ZNF143 binding sites is usually related to transcription regulation. Yang et al. (2019) have found that both active (H3K4me1, H3K4me3, and H3K27ac) and suppressive (H3K27me3) histone marks can modulate ZNF143 binding, which in turn, regulate gene expression. However, how to develop new and convenient detection systems to study the function of ZNF143 is still a big challenge. Recently, Sathyan et al. (2019) have developed an improved auxin-inducible degron system to study TF function. After rapidly depleting the ZNF143 TF, transcriptional profiling indicates that ZNF143 activates transcription in cis and regulates promoter-proximal paused RNA polymerase density.

CTCF, cohesion, and ZNF143 are three major regulators involved in the establishment and maintenance of long-range chromatin interactions. In mammalian cells, TAD-free analysis indicates that the blocking effects of CTCF, cohesin, and ZNF143 depend on the distance between loci because each protein may participate at different scales of chromatin organization (Mourad and Cuvier, 2018). CTCF and cohesin are the key factors in organizing the mammalian genome to form TADs and loops, and the CTCF loops are formed as a result of cohesin-dependent loop extrusion (Dixon et al., 2012; Nor et al., 2012; Sanborn et al., 2015; Fudenberg et al., 2016; Goloborodko et al., 2016; Busslinger et al., 2017; Nuebler et al., 2018). ZNF143 is not only involved in CTCF/cohesin-mediated chromatin interactions, but also can bind directly to the promoter and connect it to distal regulatory elements (such as enhancer) to form chromatin loops (Heidari et al., 2014; Bailey et al., 2015; Ye et al., 2016). The recurrent C→T conversion at the ZNF143 locus influences the chromatin loop formation and alters distal gene expression in breast cancer (Yang et al., 2018). Lin et al. (2017) have reported a new epigenetic feature called sparse conserved under-methylated CpGs (scUMCs) is involved in cell-specific regulation of long-range chromatin interaction mediated by chromatin-looping factors (CTCF, cohesin, and ZNF143), providing a new direction in the research of the relationship between DNA methylation and chromatin organization. Recent technical developments allow more accurately identify where TFs bind to DNA. Skene et al. have showed that their new *in situ* methods, such as cleavage under targets and release using nuclease (CUT&RUN) and cleavage under targets and tagmentation (CUT&Tag), will be viewed as a cost-effective and versatile alternative to ChIP because of low backgrounds, which requiring only ∼1/10th the sequencing depth as ChIP (Skene and Henikoff, 2017; Skene et al., 2018; Kaya-Okur et al., 2019; Meers et al., 2019). Based on these methods, Shimbo et al. (2019) have developed cleavage under tethered nuclease for conformational capture (Cut-C) technology to identify chromatin interactions mediated by a protein of interest along with the genome-wide distribution of the target proteins. Thus, using these latest technologies, we may be clearly captured the accuracy of chromatin loops mediated by ZNF143 in a genome-wide scale.

**FIGURE 2:**
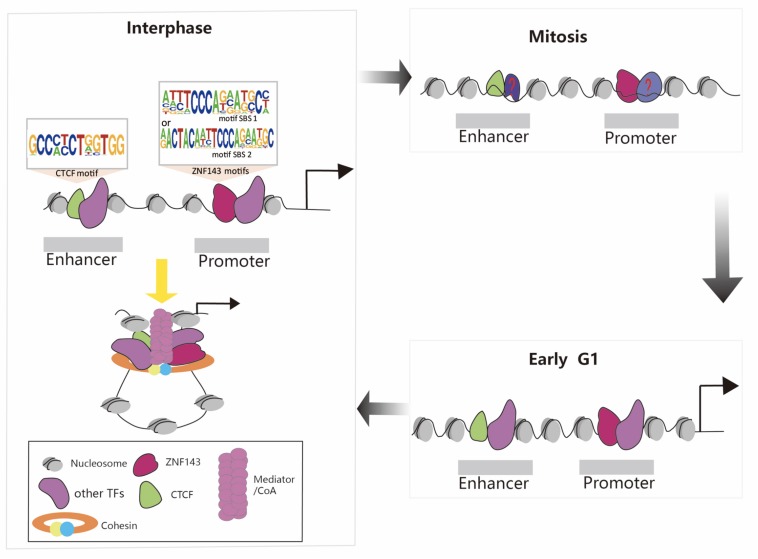
Schematic representation of chromatin loop formation mediated by ZNF143, CTCF, cohesin, and other TFs during the cell cycle. ZNF143 is a potential mitotic bookmarking factor helps to re-establish chromatin loops in early G1.

During mitosis, transcription is globally shut down, chromatin condenses, the nuclear envelope is disassembled, and most TFs are stripped off the mitotic chromosomes. How do the new daughter cells faithfully re-establish the cell-type specific transcription program? Recent discoveries that a select set of TFs remain associated with mitotic chromosomes suggest a phenomenon termed mitotic bookmarking (Huang and Wang, 2017). For example, many studies have reported that CTCF is still partially retained in mitotic chromosomes and chromatin structure dynamics during the mitosis-to-G1 phase transition (Burke et al., 2005; Yan et al., 2013; Shen et al., 2015; Teves et al., 2016; Oomen et al., 2019; Palozola et al., 2019; Zhang et al., 2019). Thus, the presence of CTCF during mitosis may function as candidate mitotic bookmarking protein. This mechanism plays a potential and critical role in maintaining cell identity and cell destiny. Meanwhile, ZNF143 can interact with CTCF and mediate the formation of the chromatin loops. We recently discovered that ZNF143 was still partially bound to the chromosome during mitosis and 80% of the retained regions preferentially localized to promoters, supporting that it functioned mainly through promoters (Ye et al., 2020). Thus, the presence of CTCF and ZNF143 during mitosis may be crucial to recruit other regulatory factors to bind to chromosomes and re-establish chromatin loops in early G1 phase (Figure 2). Therefore, further studies on ZNF143 are necessary to help reveal its regulatory mechanism during the cell cycle.

As a key TF, the role of ZNF143 in cancer progression through transcriptional regulation of genes related to DNA replication and cell cycle (Izumi et al., 2010). Furthermore, Song et al. have showed that miR-590-3p could negatively modulate the expression of ZNF143 via binding to the ZNF143 3′-UTR and ZNF143 can directly activate FAM224A expression through binding to its promoter, forming the A1CF-FAM224A-miR-590-3p-ZNF143 positive feedback loop. This loop plays a critical role in regulating the malignant progression of glioma cells, providing a novel molecular target for glioma therapy (Song et al., 2019). In recent years, with the technology and bioinformatics analysis development, the molecular mechanism of ZNF143-mediated gene transcriptional regulation has been largely exploited. Chromatin looping between promoters and distal regulatory elements depends on DNA binding by ZNF143 and other partners. In the future, how to comprehensively analyze the mechanism of ZNF143 in mediating gene expression of different cell types and discover the novel and potential functions of ZNF143 remains a considerable challenge.

## Author Contributions

BY, GaY, and YL drafted the manuscript. CZ, QW, and GuY critically revised the manuscript.

## Conflict of Interest

The authors declare that the research was conducted in the absence of any commercial or financial relationships that could be construed as a potential conflict of interest.
